# A Tumultuous Course of Exogenous Testosterone by a Bodybuilder Causing a Catastrophic Hypercoagulable State in the Surgical Intensive Care Unit

**DOI:** 10.1155/2019/3097865

**Published:** 2019-12-08

**Authors:** Kevin Lee, Sophia Toraby, Robert Cotterman, Babatunde Oriowo, John Fish

**Affiliations:** ^1^Department of Anesthesiology, The University of Toledo Medical Center, Toledo, OH, USA; ^2^Department of Surgery, The University of Toledo Medical Center, Toledo, OH, USA; ^3^Department of Critical Care and Trauma Surgery, ProMedica Toledo Hospital, Toledo, OH, USA; ^4^Department of Vascular Surgery, ProMedica Toledo Hospital, Toledo, OH, USA

## Abstract

Present literature demonstrates an equivocal relationship between testosterone and thrombogenicity. Herein, we describe a case in which a patient used an unspecified amount and duration of exogenous testosterone injections, subsequently developing thrombotic events in his: right radial artery, right iliac artery, superficial femoral artery, splenic artery and a bilateral lower lobe pulmonary embolism. As a result, clinicians should consider exogenous testosterone use as a potential risk factor when the etiology of a patient's thrombotic events are not clear. We also completed a literature review of the molecular mechanisms in which testosterone can increase the clot burden through an increases human platelet thromboxane A2 receptor density and an increase in erythropoiesis.

## 1. Introduction

The use of performance enhancing drugs (PEDs), particularly the use of androgens, is a way for some bodybuilders and nonathletes to increase muscle mass, improve performance, and enhance physical attractiveness. In fact, the use of androgenic steroids has become a global epidemic in which the overall global lifetime prevalence in men is 6.4 percent and in women is 1.6 percent [1]. The use of exogenous androgens does not come without consequences as acne, gynecomastia, liver toxicity, cardiac dysfunction, and psychiatric symptoms are known side effects which have been well documented in the literature [2]. While the association between testosterone use and venous thromboembolism in men remains dubious, we present a case in which a 32-year-old Caucasian male developed multiple venous and arterial thromboembolisms likely secondary to his exogenous testosterone use. Though there is no clear relationship between testosterone use and hypercoagulability as per a recent Systematic Review and Meta-Analysis in 2018 by Mayo Clinic, several molecular mechanisms have been proposed to explain such [3]. The first being that testosterone increases human platelet thromboxane A2 receptor density and aggregation response which could increase the thrombogenicity [4]. Secondly, testosterone stimulates erythropoiesis, which increases hemoglobin and hematocrit in a dose-dependent manner ultimately increasing the propensity of a thrombotic event [5]. As the prevalence of androgenic steroids continues to rise, further investigation for establishing testosterone as a risk factor for thrombogenicity is warranted.

## 2. Case History/Examination

**Figure 1:**
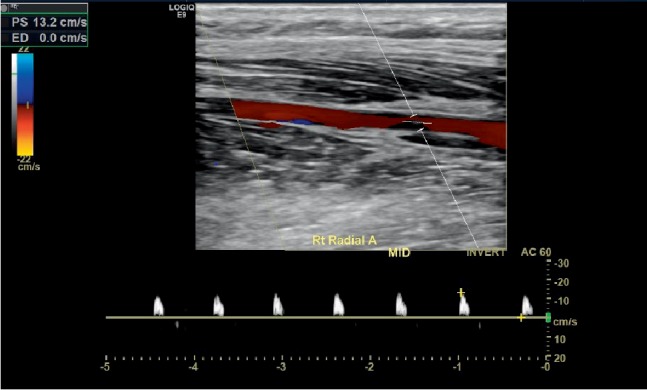
Vascular arterial duplex of the right mid radial artery with pre-occlusive “thump” waveforms. Spectral waveforms with normal triphasic diastolic flow were noted in the subclavian, axillary, brachial and ulnar artery without significant color flow disturbance.

A 32-year-old, Caucasian, morbidly-obese (BMI of 42.96 kg/m^2^), male with a past medical history of hypertension, obstructive sleep apnea was transferred from an outside facility with complaints of worsening cyanosis, paresthesias, numbness, and weakness in his right upper extremity. The patient's only home medication was 10 mg of Lisinopril daily for which his blood pressure was controlled. It should also be noted that the patient had no documented history of hyperlipidemia as a lipid profile was drawn four months prior which showed a total cholesterol level of 157 mg/dl (100−199 mg/dl), triglyceride level of 76 mg/dl (100−199 mg/dl), HDL level of 39 mg/dl (<40 mg/dl) and LDL level of 103 (<100 mg/dl). Prior to the patient's initial presentation, he had been experiencing this symptomatology three weeks prior which was managed conservatively with pain medication and steroids by his primary care physician. The patient was also fully mobilize and active placing him at minimal risk of venous stasis. At the outside facility, the patient had a CT angiogram of his right upper extremity which showed a thrombotic occlusion of the right radial artery just proximal to the wrist and a right upper extremity. Right upper extremity arterial Doppler ultrasound showed monophasic flow and was flat-line at the index finger and thumb with a preocclusive waveform in the mid radial artery and subsequent distal radial artery occlusion, as seen in Figure 1.

The patient denied any personal or family history of thrombosis. Upon review of his social history, the patient did admit to using exogenous testosterone in the past. The exact dose and duration of his testosterone use was not fully clarified with the patient directly. He also admitted to being a former smoker with an undocumented duration prior to experiencing any symptoms in his right upper extremity.

Upon presentation, the patient was started on a continuous heparin infusion and was admitted to the vascular surgery service for acute limb ischemia. The following day, the patient had a right radial artery catheterization via cut-down technique, thrombectomy of the radial artery, intra-arterial tPA into the distal radial artery and he was continued on a heparin infusion. The patient also had an echocardiogram which was negative for a cardioembolic event and the patient had no known history of cardiac arrhythmia or disease which was also not present on cardiac monitoring.

However, the next two days, the patient had developed increased swelling and pain in his right upper extremity. The orthopedic surgery team was consulted for evaluation and management for concerns of compartment syndrome and had subsequent right hand thenar and hypothenar fasciotomies. The patient also had IR angiography performed that day which showed occlusion of the mid and distal right radial artery to the level of the arch with occlusions of the interdigital arteries involving the thumb and the index finger. He was continued on a heparin infusion and started on an epoprostenol infusion. TEE was unremarkable and CBC and renal and hepatic function remained normal.

**Figure 2:**
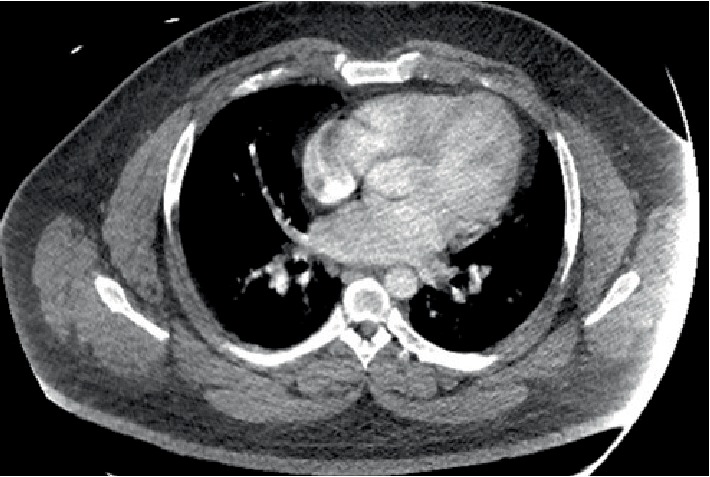
CT angiogram Chest with intravenous contrast shows small to moderate left lower lobe pulmonary emboli. A 2 cm pleural-based right lower lobe nodular density can also be seen. It should be noted that evaluation of the pulmonary arteries is suboptimal secondary to patient body habitus and under opacification of the pulmonary vessels.

**Figure 3:**
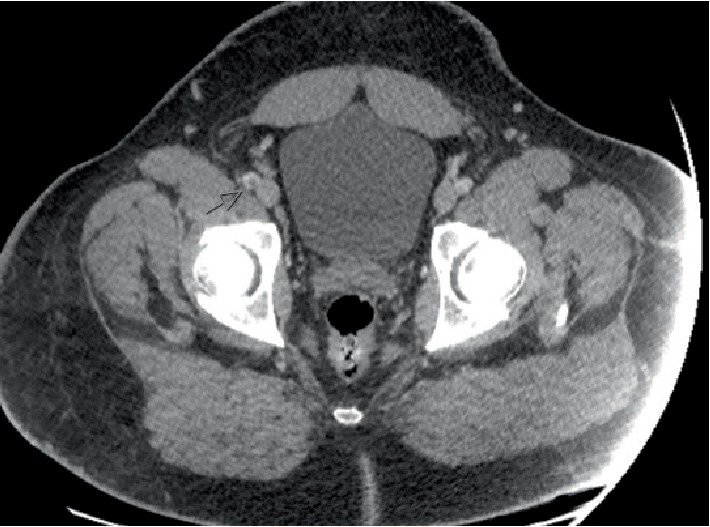
CT angiogram abdominal aorta with right lower extremity runoff shows a nonoccuslive longitudinal filling defect in the distal right external iliac artery and tibioperoneal trunk suggestive of a thrombus. There, however, are Patent common femoral artery, profunda, and SFA. Left lower extremity runoff showed a patent common femoral artery, profunda, popliteal artery and SFA. The abdominal aorta is widely patent and measures 2.3 cm the celiac, 2 cm at the SMA, and 1.7 cm below the renal arteries.

Prior to the patient being transferred to the medical-surgical floor, SICU nursing staff expressed that they could not identify a pulse in the right dorsalis pedis pulse and the extremity was cold to palpation. CTA of the chest and CTA of the abdomen were ordered and showed small to moderate, bilateral lower lobe, pulmonary emboli and a thrombus in the right external iliac artery as seen in Figures 2 and 3.

**Figure 4:**
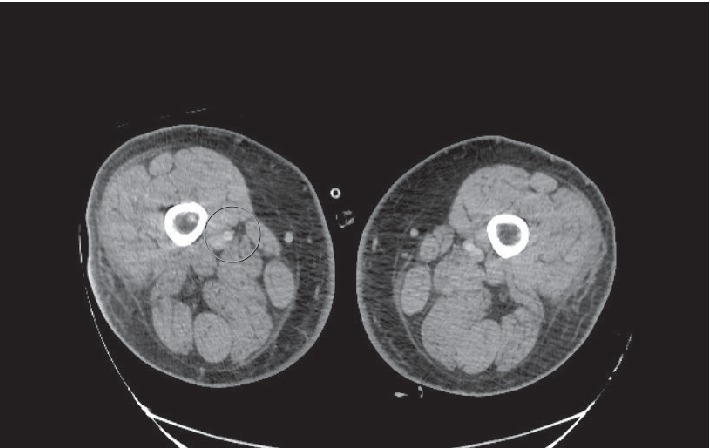
CT angiogram abdominal aorta with right lower extremity runoff shows a globular filling defect at the mid and distal segment of the right SFA most consistent with an arterial embolus. The right profunda artery is grossly unremarkable. There is grossly adequate 3 vessel runoff. Left superficial femoral artery is grossly patent as well as popliteal artery with 3 vessels runoff. Both external iliac arteries are grossly patent. Both common femoral arteries are grossly patent.

Of particular clinical significance, the CTA of the chest also showed a 2 cm pleural-based right lung nodular density. He was placed on tPA for catheter directed thrombolysis for four days in conjunction with heparin and epoprostenol. Heparin was also switched to bilvarudin due to subtherapeutic Heparin-Xa levels. The patient was not thrombocytopenic or profoundly anemic and both heparin inducted thrombocytopenia and disseminated intravascular coagulation were both ruled out. Repeated CTA of the abdomen with runoff showed a splenic infarct, and a filling defect at the mid and distal right superficial femoral artery, as seen in Figure 4, both consistent with underlying emboli.

The patient had a full hypercoagulable workup which was unremarkable and included: a hemolysis workup, homocysteine level, Heparin PF4, antiphospholipid antibodies, monoclonal protein, paroxysmal nocturnal hemoglobinuria, JAK2 mutation, Factor V Leiden mutation, and Prothrombin mutation as seen in Table 1. Initial testosterone and luteinizing hormone levels were not drawn on presentation or during admission as this was a diagnosis of exclusion, at the time, because of the lack of clear relationship between testosterone use and hypercoagulability in previous studies. The decision was made to not order Protein C, Protein S, Antithrombin 3, and lupus anticoagulant studies, per the oncology team, because the patient was already on anticoagulation and the values would be confounding.

During the patient's hospital course, the patient also developed septic shock secondary to *A. baumannii* complex, aspiration pneumonia, and C diff colitis. He went into Acute Respiratory Distress Syndrome as a manifestation of his septic shock and eventually required tracheostomy and percutaneous endoscopic gastrostomy due to severe decompensated respiratory status.

Upon improvement of the patient's clinical status, he was transitioned to a long-term acute care facility. He was discharged on warfarin with INR goal of 2.5–3.5, ticagrelor and baby aspirin and he was instructed to follow up with vascular medicine as an outpatient. Despite, no clear etiology for the patient's hypercoagulable state, it is suspected that the patient's exogenous testosterone use prompted his profound hypercoagulable state among other risk factors. It should be noted that the patient did initially present with a hematocrit of 51.6%. As an outpatient, the patient continued to be evaluated by vascular medicine for thrombophilia, which was negative for standard genetic and acquired conditions. Presently, the patient symptomatically has some mild residual weakness in his right upper and lower extremities.

## 3. Discussion

While there have been previous documented case reports that have highlighted an association between exogenous testosterone use and pulmonary embolus, we present a unique case in which our patient developed diffuse multi vessel arterial and venous thrombotic events [6]. During our patient's 43 day hospital course, we treated the patient for thrombotic events in his right radial artery, right iliac artery, superficial femoral artery, splenic artery, and a bilateral lower lobe pulmonary emboli. The patient did have several risk factors to develop a thrombotic event including obesity and a smoking history, however, the acuity and recurrence of thrombotic events in a short timeframe make these risk factors an unlikely sole contributor. The patient did have a 2 cm pleural-based right lung nodular density seen on imaging. Given the presence of multi organ failure during this patient's hospital admission, it was recommended by our hematology/oncology team that this be evaluated further as an outpatient. Given the negative hypercoagulable workup, we felt that the patient's clot burden was most likely associated with his recent exogenous testosterone use as he did not present with any other plausible risk factors.

Multiple studies have not established a clear relationship between thrombogenicity and exogenous testosterone use [7]. Thromboxane A_2_ (TXA_2_), a major arachidonic acid metabolite of platelets, acts through membrane surface receptors to aggregate platelets and constrict vascular smooth muscle. A study done by Ajayi, et al. demonstrated that testosterone increases platelet TXA_2 _receptor density and aggregation responses in both in vitro and in vivo studies. Specifically, they found that testosterone treatment significantly increased the human platelet TXA_2_ receptor density with a peak effect at 4 weeks and a return to baseline at 8 weeks [4]. Further, results show that the increase in platelet TXA_2_ receptor density was significantly (*P* < 0.001) greater in the testosterone-treated group than in the placebo group or its own baseline. Given the timeframe that Ajayi, et al. [4] established between testosterone and peak effect of TXA_2_ receptor, it would be reasonable to suspect that our patient's testosterone use for five weeks prior would have placed him at a greater propensity toward clot formation.

Testosterone has also been well documented in the literature to stimulate erythropoiesis. A study done by Bachman, et al. demonstrates that increases in hemoglobin and hematocrit are due to testosterone stimulating erythropoietin (EPO) and reducing ferritin and hepcidin concentrations. As a result, this recalibrates the set point of EPO in relation to hemoglobin and by increasing iron utilization for erythropoiesis [5]. The authors found that the testosterone plays two pivotal roles in increasing red cell mass. First, the testosterone inhibits BMP-Smad signaling in hepatocytes leading to suppression of hepcidin transcription [5]. Secondly, the testosterone stimulates renal secretion of EPO, stimulating erythropoiesis which could then further suppress hepcidin. This causes a cascade which subsequently upregulates the expression of GATA-1- and GATA-dependent genes, which could also increase EPO sensitivity and stimulate stress erythroposis [5]. Testosterone is then converted to estradiol and can regulate hepcidin transcription. In our patient, his serum ferritin level (12–300 ng/mL) was elevated to 668 ng/mL and his erythropoietin level (2.6−18.5 mIU/mL) was 104.4 mIU/mL which was drawn approximately two weeks later. A potential explanation for an elevated ferritin level seen in our patient as opposed to a decreased ferritin level as Bachman, et al. describes in their cascade is that the ferritin level was drawn early in the patient's hospital course. Therefore, the dualistic stimulation of the patient's platelets through the TXA_2_ pathway and increased erythropoiesis likely explains this patient's arterial thrombi.

In regards to the underlying mechanism for the patient's venous thromboembolism, it can certainly be explained by a provoked event related to the patient's ICU stay and sepsis. However, more interestingly, we propose that the patient's exogenous testosterone use may have had an undescribed effect on the activation of clotting factors. The patient was discharged on warfarin with an INR goal of 2.5–3.5, ticagrelor and baby aspirin but required an INR as high as 4.32 in order to achieve Factor II and Factor X suppression.  Table 2 is a correlation between the INR levels and Factor II and Factor X levels. The decision to manage the patient initially with an INR of 2.5–3.5 was made because the patient's factor inhibition on warfarin within the INR range of 2.0–3.0 was not adequate in the less than 30% range, despite having a negative mixing study and lupus anticoagulant or antiphospholipid antibodies. Further, platelet mapping with thromboelastography (TEG) was of particular clinical benefit to assist us in guiding the choice of antiplatelet medications and efficacy. The TEG results with R-time of 12.2 min, K-time of 1.5 min and a-angle of 69.0 for our patient are shown, which showed sufficiency with decreasing clot burden while on ticagrelor.

While there is an inherent risk for vascular events due to testosterone use, this association needs to be further elucidated with additional studies [8]. With the rise of exogenous testosterone use for performance enhancing reasons, particularly those that are not approved by the FDA, our case report demonstrates such potential risks and consequences, especially since the purity of the injected androgenic substance also could not be verified. Vigilance for testosterone as a potential risk factor for thrombogenicity is essential in patients with an unclear etiology as this can contribute to improved outcome in patients.

## Figures and Tables

**Table 1:** Initial hematologic studies which indicate an unremarkable coagulopathic evaluation.

Initial hematologic labs	Reference range and units	Result
Hemoglobin	13.1–17.3 g/dl	15.0
Hematocrit	39–49%	44.4
Platelets	150–450 × 10^9^/L	330
Heparin anti-xa unfractioned	0.30–0.70 IU/mL	0.36
Protime	9.5–12.6 sec	12.3
Inr	0.9–1.2	1.1
Anticardiolipin IgA	0–19.9 APL	0.5
Anticardiolipin IgM Ab	0–19.9 MPL	0.5
Anticardiolipin IgG	0–19.9 GPL	<1.6
Beta-2 gp1 IgA	0–19.9 u/mL	<0.6
Beta-2 gp1 IgM	0–19.9 u/mL	0.5
Beta-2 gp1 IgG	0–19.9 u/mL	<1.4
Homocysteine	5.90–16.00 mcmol/L	14.64
Collagen/epinipherine	0–179 sec	112
Collagen/adp	0–114 sec	91
Heparin pf4 antibody (hit)	<0.4 OD	0.168
JAK2 exon 12 mutation detection	—	Negative. No pathogenic genetic alterations were detected in JAK2, exons 12–15.
Pnh by flow cytometry	—	No evidence of paroxysmal nocturnal hemoglobinuria detected by flow cytometry.
Factor V leiden R506Q gene mutation		Negative. The patient does not have the Factor V leiden mutation.
Prothrombin G20210A gene mutation		Negative. The patient does not have the prothrombin G20210A mutation.

**Table 2:** Correlation between INR levels and Factor X and Factor II levels as a manifestation of an undescribed effect from exogenous testosterone use.

	Reference range and units	3/15/19	4/18/19
Protime	9.5–12.6 sec	18.9	37.4
Inr	0.9–1.2	1.6	3.2
Factor X activity	70–150% act	93	11
Factor II activity	79–131% act	63	29
